# Applications and advancements of CRISPR-Cas in the treatment of lung cancer

**DOI:** 10.3389/fcell.2023.1295084

**Published:** 2023-12-11

**Authors:** Pan Lei, Yixin Ju, Fenfen Peng, Jie Luo

**Affiliations:** ^1^ Hubei Clinical Research Center for Precise Diagnosis and Treatment of Liver Cancer, Taihe Hospital, Hubei University of Medicine, Shiyan, Hubei, China; ^2^ Hubei Hongshan Laboratory, College of Biomedicine and Health, Huazhong Agricultural University, Wuhan, China; ^3^ Department of Pharmacy, Jianyang City Hospital of Traditional Chinese Medicine, Chengdu University of Traditional Chinese Medicine, Jianyang, Sichuan, China; ^4^ Department of Neurosurgery, Taihe Hospital, Hubei University of Medicine, Shiyan, Hubei, China

**Keywords:** CRISPR/Cas, lung cancer, diagnosis, treatment, Tuba-seq, mouse model, delivery method

## Abstract

Lung cancer is one of the most malignant diseases and a major contributor to cancer-related deaths worldwide due to the deficiency of early diagnosis and effective therapy that are of great importance for patient prognosis and quality of life. Over the past decade, the advent of clustered regularly interspaced short palindromic repeats/CRISPR associated protein (CRISPR/Cas) system has significantly propelled the progress of both fundamental research and clinical trials of lung cancer. In this review, we review the current applications of the CRISPR/Cas system in diagnosis, target identification, and treatment resistance of lung cancer. Furthermore, we summarize the development of lung cancer animal models and delivery methods based on CRISPR system, providing novel insights into clinical diagnosis and treatment strategies of lung cancer.

## 1 Introduction

**FIGURE 1:**
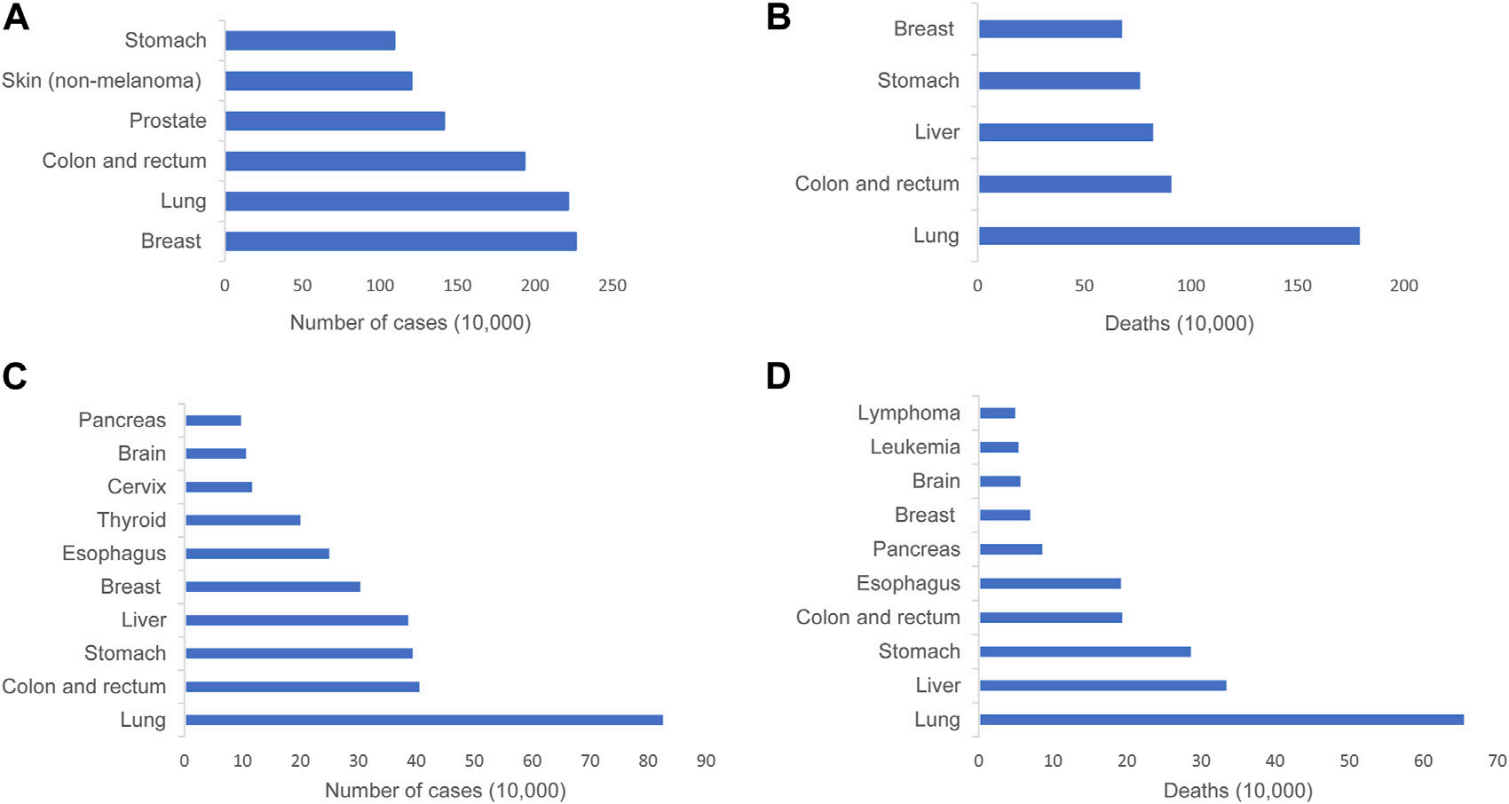
Graphical representation depicting the incidence and mortality rates of lung cancer across the globe and in China. **(A)** Incidence of the most common cancers worldwide in 2020. **(B)** The most common causes of cancer death worldwide in 2020. **(C)** Incidence of top 10 malignant tumors in China, 2016. **(D)** Top 10 malignant tumor deaths in China, 2016..

Lung cancer exhibits the highest global mortality rate compared to other types of cancer, particularly in China, where both the incidence and mortality rates of lung cancer rank the first (Figure 1) (World Health Organization, 2023; Zheng et al., 2023) (Supplementary Data Sheet 1). Over the recent decades, the advancements in modern technologies and the growing comprehension of the intrinsic patterns of diseases have resulted in a decrease in the mortality rate of lung cancer. However, numerous patients exhibit undesirable prognoses and compromised quality of life due to the lack of early diagnosis and timely effective intervention (Gridelli et al., 2015; Thai et al., 2021; Siegel et al., 2023). A great number of lung cancer biomarkers have been identified such as *EGFR* mutations, *ALK* and *ROS-1* rearrangements, and HER-2 overexpression. The discovery of the predictive biomarkers promotes therapeutic decisions at the time of diagnosis and during disease progression. But targeted therapies based on the biomarkers inevitably occur resistance, although patients harbor targetable mutated genes (Gridelli et al., 2015; Thai et al., 2021). Therefore, further enhancement of the understanding of lung cancer biology is needed in order to develop more robust predictive biomarkers and more efficacious treatment methods.

Clustered regularly interspaced short palindromic repeats (CRISPR) and CRISPR-associated (Cas) proteins have been identified as mediators of adaptive immunity in prokaryotes, mediating resistance to viral infections and exogenous nucleic acid invasions (Barrangou et al., 2007; Brouns et al., 2008). The spacer regions and upstream part of CRISPR locus are transcribed to produce pre-crRNA and tracrRNA respectively. Subsequently, the tracrRNA:crRNA complex directs the Cas9 nuclease to generate a double-stranded break (DSB) at the target sequence (Jinek et al., 2012; Wang et al., 2022). The invading nucleic acid sequences are integrated into the CRISPR locus as spacer regions, thus allowing the CRISPR/Cas system to resist re-invasion by viruses or plasmids under the guidance of crRNA (Wang et al., 2022). Based on this, a single guide RNA (sgRNA) can be artificially engineered to replace the tracrRNA:crRNA complex, enabling targeted cleavage of any PAM-containing DNA (Jinek et al., 2012). In addition to Cas9-mediated gene silencing, the CRISPR system can also modulate target gene expression at the transcriptional level (Gilbert et al., 2013), which greatly expands the applications of CRISPR technology in tumor.

**FIGURE 2:**
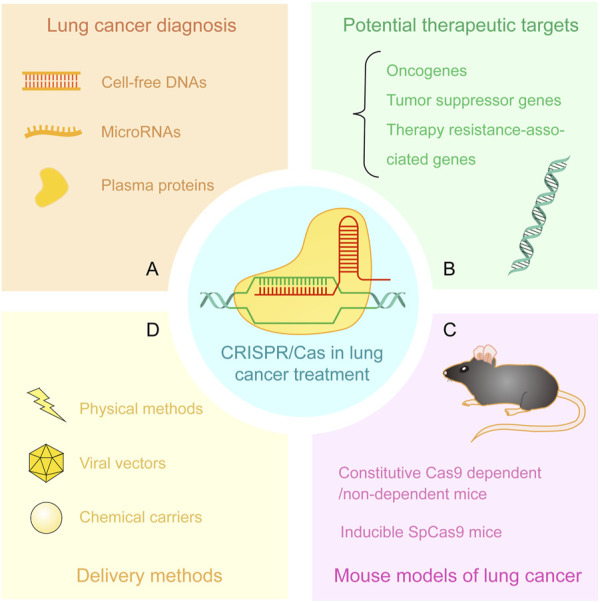
Applications of CRISPR/Cas system in lung cancer treatment. **(A)** Detection of cell-free DNAs, microRNAs, and plasma proteins from patient samples by CRISPR/Cas-based approaches. **(B)** Discovery and verification of potential therapeutic targets including oncogenes, tumor suppressor genes, and therapy resistance-associated genes. **(C)** CRISPR-based mouse models of lung cancer. **(D)** The delivery methods of CRISPR/Cas components including physical methods, viral vectors, and chemical carriers.

Compared to conventional gene editing techniques such as zinc-finger endonucleases (ZFNs) and transcription activator-like effector nucleases (TALENs), the straightforward and precise CRISPR/Cas system exhibits more potent functions in gene editing and finds more extensive applications in cancer biology and therapy such as modeling mutational heterogeneity, identifying therapy vulnerabilities, and clinical care (Katti et al., 2022). In this review, we summarize the recent advancements of CRISPR technology in the diagnosis, exploration of intrinsic mechanisms, and discovery of potential therapeutic targets of lung cancer. We review the applications of CRISPR/Cas system in the establishment of lung cancer animal models and delivery methods of CRISPR vectors (Figure 2).

## 2 Applications of CRISPR in diagnosis for lung cancer

The absence of early and accurate diagnosis is the leading cause of a poor prognosis of patients with lung cancer (Gridelli et al., 2015). To timely prevent the malignant development of lung cancer and improve patient prognosis, there is a continued need for further exploration of effective and rapid diagnostic methods.

Cell-free DNA (cfDNA) carrying oncogenic mutations is emerging as a novel biomarker for disease monitoring and guiding clinical interventions. However, it is still a challenge to develop general methods with a high signal-to-noise ratio to detect rare oncogenic mutated alleles in clinic (Aalipour et al., 2018). The precise targeting capacity of the CRISPR/Cas system can be used to develop the techniques for detecting rare disease-specific mutations. The first platform combining CRISPR-deactivated Cas9 (dCas9), magnetic beads, and allele-specific qPCR was set up to test the most common *EGFR* mutations including exon 19 deletion, T790M, and L858R in cfDNA samples of patients with non-small cell lung cancer (NSCLC) (Aalipour et al., 2018). The establishment of this platform resulted in more than 20-fold increases in mutated allele frequency compared with detecting these mutations by qPCR at an allele frequency of 0.1%. Another technology combining CRISPR system and post-PCR cfDNA (CRISPR-CPPC) was developed to detect the T790M mutation of *EGFR* gene. Based on clinical diagnosis, CRISPR-CPPC technology achieved a sensitivity of 93.9% and a specificity of 100% in detecting the T790M mutation (Kim et al., 2022). The diagnostic ability of CRISPR-CPPC is superior to that of any other available strategy in testing patients with progressive disease.

MiRNAs play a potential function in cancer diagnosis, and they are beneficial for the clinical management of diseases (Jiang et al., 2019). The method combining rolling circle amplification (RCA) and dCas9-split horseradish peroxidase (HRP) fusion protein can robustly detect circulating miRNAs such as let-7a, which was found to have a significantly lower expression in NSCLC patients than in healthy volunteers (Qiu et al., 2018). Thus, this method can distinguish patients with NSCLC from health people. Another method combining RCA, CRISPR/Cas9, and FAM fluorescence showed single-base resolution in detecting extracellular vesicle-derived microRNAs from both cultured cells and clinical patients (Wang et al., 2020). Recently, a miRNA detection approach was developed based on RCA, CRISPR/Cas9, and catalytic hairpin assembly (CHA) technologies, and this approach was used to analyze clinical serum samples. The results showed significant differences (*p* < 0.001, *n* = 10) between lung cancer patients with and without brain metastasis (Liu et al., 2022). In addition to Cas9, other Cas proteins such as Cas12a and Cas13a, have been employed for the diagnosis of lung cancer. One combinatory method (CRISPR/Cas12a + magnetic nanoparticles + cascade strand displacement reaction (CSDR)) was established, and this method exhibited ultra-high sensitivity and specificity in detecting exosomal miR-21 in blood samples from lung cancer patients. The last fluorescent biosensor of the work flow enables the distinguishment of lung cancer patients from healthy individuals by determining miR-21 (Liu et al., 2022). Overexpression of miR-944 and miR-205 in lung cancer patients can also be sensitively detected using an AND logic-gate-based CRISPR-Cas12a biosensing platform with magnetic beads and glucose oxidase (Gong et al., 2022), and the application of this platform achieves the simultaneous detection of multiple markers within a single assay. Furthermore, a novel approach combining CRISPR/Cas13a, a catalytic hairpin DNA circuit (CHDC), and a reusable electrochemical biosensor has been established to detect NSCLC-associated RNAs. This method enables the sensitive, rapid, and precise detection of RNAs including miR-17, miR-155, TTF-1 mRNA, miR-19b, miR-210, and EGFR mRNA in patient serum (Sheng et al., 2021).

In addition to nucleic acids from patients, plasma proteins are also biomarkers associated with cancer diagnosis (Landegren and Hammond, 2021). Human 8-oxoguanine DNA glycosylase (hOGG1) is relative to DNA oxidative damage and repair, and flap structure-specific endonuclease 1 (FEN1) plays a crucial role in DNA replication and cell proliferation, both of which are promising biomarkers in lung cancer. Although there are some methods to measure them, the methods did not meet the requirement of clinical accurate detection. Currently, a novel platform using DNA dumbbell probes, rolling circle transcription (RCT), and CRISPR/Cas12a technology has been developed for the simultaneous detection of hOGG1 and FEN1 in human serum samples (Cheng et al., 2023). Moreover, the combination of DNAzyme walkers and CRISPR-Cas12a/Cas13a systems was used to simultaneously detect exosomal protein biomarkers of lung cancer, serum amyloid A-1 protein (SAA1), and coagulation factor V (FV), exhibiting high sensitivity (30.00 pg/mL for SAA1 and 200.00 pg/mL for FV), specificity, and accuracy (86.96%) (Ding et al., 2022). With the further advancement of technologies, these detections based on CRISPR/Cas system will play a more powerful role in early screening and diagnosis of lung cancer as well as the prognosis assessment.

## 3 Identification of potential therapeutic targets of lung cancer

In addition to its role in assisting with the diagnosis of lung cancer, CRISPR also serves as a crucial and powerful tool for identifying and validating cancer-associated genes, including oncogenes, tumor suppressor genes, and therapy resistance-related genes (Jiang et al., 2019; Liu et al., 2023). As the potential therapeutic targets, the exploration of these genetic mechanisms will promote the progress of new effective targeted strategies in clinical treatment, ultimately improving patient prognosis and quality of life. Considering this, we will review the studies of intrinsic mechanisms underlying lung cancer tumorigenesis, development, therapy resistance based on CRISPR gene editing technology, focusing on lung cancer-associated genes. In addition, we describe a new CRISPR technology combined with tumor barcode sequencing and highlight its promise in lung cancer research.

### 3.1 Oncogenes

Oncogenes derived from mutated proto-oncogenes disrupt normal balanced states and give rise to excessive cell proliferation, malignant transformation of normal cells, and immune evasion (Bell, 1988; Petroni et al., 2022). Targeted therapy for oncogenes is the standard first-line treatment to patients with validated genetic alterations in advanced-stage NSCLC (Gridelli et al., 2015). However, this therapy will invariably produce resistance, consequently failing to slow down and stop disease progression (Gridelli et al., 2015; Thai et al., 2021). Deciphering the mechanisms of tumorigenesis and development and combating therapy resistance remain a formidable challenge. CRISPR gene editing system can precisely recognize genome sequence, thus efficiently editing targeted gene and modulating gene transcription (Gilbert et al., 2013; Hsu et al., 2013; Ran et al., 2013), which provides a powerful tool to investigate the malignant disease. CRISPR technology has been successfully applied to implement the editing and modulation of numerous oncogenes such as epidermal growth factor receptor (*EGFR*), *KRAS*, focal adhesion kinase (*FAK*), metabotropic glutamate receptor 8 (*GRM8*), *SMAD3/4*, and *MET* in lung cancer (Table 1).

**TABLE 1:** CRISPR technology in functional studies for genes concerned with tumor initiation and development of lung cancer.

Targets	Knockout/other	Edited objects	Outcomes after editing	Cancer types	Role	References
*EGFR* L858R	Knockout	H1975 and A549 cells; xenograft mouse model of human lung cancer	Inhibition of tumor growth and significant decrease of tumor size in xenograft mouse model	LUAD	OG	Koo et al. (2017)
*EGFR* L858R	Knockout	H1650 cells	Reduced cell proliferation; decreased tumor load *in vivo*	LUAD	OG	Cheung et al. (2018)
*KRAS* G12S	Knockout and knockdown	A549 and H2228 cells; xenograft mouse models of 2 cell lines	Retarded cell proliferation and tumor growth	LUAD	OG	Gao et al. (2020)
*FAK*	Knockout	Mutant *KRAS* cells (A549 and H460 cells)	Sustainable DNA damage and susceptibility to radiotherapy	NSCLC	OG	Tang et al. (2016)
*HDAC2*	rs13213007	HEK293 cells	Facilitated cell proliferation, migration, and invasion *in vitro* by increasing c-Myc and cyclin D1 levels	NSCLC	OG	Wang et al. (2023a)
	Base editing					
*RSF1*	Knockout	H460 and H1299 cells	G1 phase arrest; increased cell apoptosis; decreased migration and cell proliferation	LC	OG	Chen et al. (2017)
*CD38*	Knockout	A549 cells and xenograft mouse model	Inhibited cell growth, invasion and xenograft growth in nude mice	LUAD	OG	Bu et al. (2018)
*CTNND2*	Knockout	Lewis lung cells and xenograft in C57BL/6 mice	Depletion of δ-catenin proteins; Loss of tumorigenic and metastatic abilities *in vivo*	LUAD	OG	Huang et al. (2018)
*GRM8*	Activation	EBC-1 and SK-MES-1 cells	Enhanced cell proliferation by suppressing the PKA activities and activating MAPK pathway	LUSC	OG	Zhang et al. (2019)
	A112G Point mutation	293T cells				
*ABCG2*	Knockout	A549 cells	EGFP knock-in generating ABCG2 knockout and *in situ* tagged ABCG2 reporter cells, which dissects the expression pattern of ABCG2 under medications such as HDAC inhibitors, hypoxia-mimicking agents and glucocorticoids	LUAD	OG	Kovacsics et al. (2020)
*SMAD3/SMAD4*	Knockout	NSCLC cells	Impaired TGF-β-induced EMT and metastasis through reducing MYOCD mRNA expression	NSCLC	OG	Tong et al. (2020)
*CAMKK1*	rs7214723 T > C mutation	A549 and H358 cells	Significantly inhibited cell proliferation and migration; promoted cell apoptosis	LUAD	OG	Zhang et al. (2021)
*YAP1*	Knockout	H69AR cells; xenograft in *Rag2* ^*−/−*^: *Jak3* ^*−/−*^ mice	Suppressed cell proliferation and migration	SCLC	OG	Saito et al. (2022)
*IGF1R*	Knockout	HCC827 cells	Amplification of MET gene; increased epithelial signature	LUAD	OG	Hussmann et al. (2017)
*MET*	Exon 14 depletion	H292, H125 and A549 cells; xenograft mouse model	Boosted migration and metastasis *in vivo*; Significantly increased tumor growth than MET	NSCLC	OG	Wang et al. (2022a)
*MFN2*	Knockout	A549 cells; tumor xenograft model	Enhanced cell viability, colony formation, and invasion *in vitro* and *in vivo*	LUAD	TS	Xu et al. (2017)
*KEAP1*	Knockout	A mouse model of *KRAS*-driven LUAD	Hyperactivation of NRF2; facilitating KRAS-driven LUAD in mice	LUAD	TS	Romero et al. (2017)
miR-1304	Knockout	A549 cells	Promoted tumor growth by increasing the protein and mRNA expression level of heme oxygenase-1	LUAD	TS	Li et al. (2017)
*GOT1*	Knockout	A549 cells	Induced cell death upon glucose deprivation	LUAD	TS	Zhou et al. (2018)
*SIK1, SIK2*	Knockout	*Kras*-driven mouse model of LUAD	Rapid lung tumor growth	LUAD	TS	Murray et al. (2019)
*PTEN*	Knockout	A549 and H460 cells; xenograft model in nude mice	Faster growth, migration, invasion and higher metastatic potential *in vivo* and vitro	NSCLC	TS	Perumal et al. (2019)
*FGA*	Knockout	H1299 and A549 cells; A549 xenograft model	Facilitated tumor growth and metastasis via integrin-AKT pathway	NSCLC	TS	Wang et al. (2020b)
*USP15*	Knockout	H1299 and A549 cells	Increases of cancer migration and invasion by TRAF6-BECN1 signaling axis in response to TLR4 stimulation	NSCLC	TS	Kim et al. (2022b)
*HPGDS*	Knockout	A549 cells	Enhanced lipid synthesis and invasion	LUAD	TS	Shoa et al. (2022)

CRISPR/Cas9-based approach has been employed to knock out mutant *EGFR*, resulting in the inhibition of proliferation of lung adenocarcinoma (LUAD) cell lines and significant reduction in tumor size and weight in xenograft mouse models (Koo et al., 2017; Cheung et al., 2018). Knockout of *EGFR* by CRISPR/Cas9 system has become a potential strategy to resensitize EGFR-targeted therapy and avoid acquired resistance caused by secondary mutations in this gene. Knockout or knockdown of *KRAS* G12S mutant allele inhibits proliferation of A549 cell line harboring the *KRAS* G12S mutation and tumor growth of xenograft models of A549 cells (Gao et al., 2020). *FAK* gene is overexpressed in several cancer types and modulate cytoskeleton remodeling, migration and apoptosis resistance. Silencing *FAK* gene can result in the inhibition of clonogenic capacity of large-cell lung carcinoma (LC) cells H460, constant DNA damage, and sensitivity enhancement to ionizing radiation (Tang et al., 2016). Moreover, *MET* exon 14 depletion by employing CRISPR editing technology leads to the enhanced cell migration, tumor invasion, and tumor metastasis through the HGF/MET axis in NSCLC. This investigation supports that ablation of MET kinase could be a promising therapeutic strategy to the patients with NSCLC and *MET* exon 14 skipping (Wang et al., 2022). Furthermore, the application of the CRISPR system to mediating transcriptional activation and the A112G point mutation confirmed the role of *GRM8* in promoting cell proliferation in squamous cell lung carcinoma (LUSC) (Zhang et al., 2019). In addition, silencing SMAD3/SMAD4 downstream of TGF-β leads to the downregulation of Myocardin (MYOCD) mRNA expression, subsequently impedes TGF-β-induced epithelial-mesenchymal transition (EMT) and metastasis in NSCLC, indicating the correlation between MYOCD and SMAD3/SMAD4 in TGF-β induced EMT (Tong et al., 2020). The feasibility and effectiveness of CRISPR technology in the targeted knockout and expression regulation of oncogenes have been sufficiently demonstrated, and this technology will further promote the development of functional validation and therapeutic research on oncogenes.

### 3.2 Tumor suppressor genes

Like oncogenes, tumor suppressor genes play a pivotal role in the initiation and progression of cancer. The loss and inactivation mutations of tumor suppressor genes will result in cell uncontrolled division, thus inducing tumorigenesis and cancer formation (National Human Genome Research Institute, 2023). Under the help of CRISPR/Cas9 gene editing technology, many properties and functions of tumor-suppressor genes have been characterized such as Kelch-like ECH-associated protein 1 (*KEAP1*), ubiquitin-specific peptidase 15 (*USP15*), *LKB1*, and members of *SIK* family (Table 1), which will provide a solid foundation for treatment of patients with identified aberrations in lung cancer.

In *KRAS*-driven LUAD, *KEAP1* deletion by CRISPR/Cas9 system hyperactivated nuclear factor erythroid 2-like 2 (*NRF2*) and accelerated lung tumorigenesis in glutaminolysis-dependent manner (Romero et al., 2017). Ablation of LKB1 and its substrates SIK1/3 revealed their tumor suppressive effect *in vivo* (Murray et al., 2019). Additionally, microRNA-1304 (miR-1304) knockout by CRISPR/Cas9 technology promoted A549 LUAD cell proliferation by upregulating heme oxygenase-1 at both protein and mRNA levels (Li et al., 2017). After knocking out deubiquitinating enzyme *USP15* by CRISPR technology, TLR4 stimulation induces autophagy of NSCLC cell lines, thus increasing their migration and invasion, and *USP15* is identified as a tumor suppressor in lung cancer progression (Kim et al., 2022). Furthermore, the knockout of phosphatase and tensin homolog (*PTEN*) or fibrinogen alpha (*FGA*) by CRISPR/Cas9 enhanced proliferation, migration, and invasion of NSCLC cells, and facilitated tumor growth and metastasis in xenograft models of immune-deficient mice (Perumal et al., 2019; Wang et al., 2020).

After identifying these tumor suppressor genes in lung cancer, targeted agonists could be developed and employed or gene editing for repairing impaired or mutated tumor suppressor genes to treat patients.

### 3.3 Therapy resistance-associated genes

As aforementioned, the resistance to targeted therapy is always inevitably produced in almost all patients with lung cancer. The mechanisms of resistance to gene-targeted therapies include oncogene amplification, secondary mutations, and activation of downstream or bypass pathways (Westover et al., 2018). Similar to gene-targeted therapy, the development of resistance to chemotherapy is affected by multiple factors such as the changes of cell cycle and apoptosis-associated factors (Stewart, 2010). Target gene editing based on CRISPR technology has provided substantial evidence for the mechanisms underlying chemoresistance and desensitization to targeted therapies (Table 2).

**TABLE 2:** CRISPR in researches related to therapy resistance-associated genes in lung cancer.

Targets	Knockout/other	Edited objects	Outcomes	Therapeutics (sensitivity)	Cancer types	References
*MET*	Exon 14 deletion	HEK293 cells	Increased cellular growth and MET inhibitor sensitivity	Crizotinib (+)	LUAD	Togashi et al. (2015)
*MAP2K1*	Knockout	H1437 cells	Decrease in cell viability; enhanced sensitivity to MEKi including trametinib	Trametinib (+)	LUAD	Gannon et al. (2016)
*SLFN11*	Knockout	Patient-derived xenograft model of SCLC	Conferred resistance to PARPi	Talazoparib (−)	SCLC	Lok et al. (2017)
*RSF-1*	Knockout	H460 and H1299 cellls; H460 cell xenograft mice	Reduced cell proliferation and migration; increased apoptosis and sensitivity to paclitaxel	Paclitaxel (+)	LC	Chen et al. (2017)
miR-214	Knockout	HCC827 cells	Reversed erlotinib resistance by upregulating LHX6	Erlotinib (+)	LUAD	Liao et al. (2017)
*KEAP1*	Knockout	CALU1, HCC364, HCC827 and MGH065 cells	Impaired sensitivity to multiple drugs	Trametinib (−), vemurafenib (−), erlotinib (−), LDK378 (−)	NSCLC	Krall et al. (2017)
*IGF1R*	Knockout	HCC827 cells	Acquired erlotinib resistance through MET-amplification	Erlotinib (−)	LUAD	Hussmann et al. (2017)
*ERCC1*	Knockout	NSCLC cell lines	Hypersensitized cells to cisplatin in p53 WT cells	Cisplatin (+)	NSCLC	Heyza et al. (2019)
β-catenin	Knockout	A549 cells	Resenstization to paclitaxel through miR-421/KEAP1 axis	Paclitaxel (+)	LUAD	Duan et al. (2019)
*p53*	Domain depletion	A549 cells	Significantly increase in cell proliferation; changed sensitivity to PI3Ki	PI3K inhibitors	LUAD	Hou et al. (2020)
*INPP4B*	Knockout	A549 cells	Led to sensitization to ionizing radiation (IR), PARP inhibitor olaparib and impaired DNA damage repair	IR (+); olaparib (+)	LUAD	Sun et al. (2020)
*PI3K*	Knockout	H460 cells	Reestablished the drug sensitivity including mitoxantrane	Mitoxantrane (+)	LC	Zhang et al. (2020)
*RSK4*	Knockout	A549 cells; xenograft model in nude mice	Reduced tumor growth and decreased cisplatin resistance	Cisplatin (+)	LUAD	Chrysostomou et al. (2021)
*NRF2*	Gene editing	A549 cells	Increased sensitivity to cisplatin	Cisplatin (+)	LUAD	Banas et al. (2022)
	Knockout	A549 cells and xenograft mouse models	Slower cell proliferation; Increased chemosensitivity	Cisplatin and carboplatin (+)	LUAD	Bialk et al. (2018)
*YAP1*	Knockout	H69AR cells	Reacquired drug sensitivity in multidrug resistance H69AR cells	Etoposide (+)	SCLC	Saito et al. (2022)
*RBMS3*	Knockout	*BRAF* ^*V600E*^ GEM model	Promoted resistance to dabrafenib plus trametinib	Dabrafenib plus trametinib (−)	NSCLC	Vaishnavi et al. (2022)
*NNT*	Methylation editing	A549 cells	Rescued the cisplatin resistance by reducing autophagy	Cisplatin (+)	LUAD	Xu et al. (2023)
*CASD1*	Knockout	A549 cells	Enhanced cell proliferation and conferred resistance to mitoxantrone	Mitoxantrone (−)	LUAD	Tuffour et al. (2023)
*CCDC6-RET, ESYT2-BRAF, FGFR3-TACC3, EML4-ALK*	Gene editing	PC9 cells	Validated their resistant mechanisms to osimertinib	Osimertinib (−)	LUAD	Kobayashi et al. (2022)
*AGK-BRAF*	Gene editing	H1975, PC9 and HCC827 cells	Led to resistance to osimertinib by increasing phosphorylation of BRAF, MEK1/2, ERK1/2, and STAT3	Osimertinib (−)	LUAD	Vojnic et al. (2022)
*EZR-ROS1*	Gene editing	HBECs	Introduced resistance to ROS1 TKIs by activating MAPK pathway	ROS1 TKIs (−)	LUAD	Sato et al. (2020)

Knockout of remodeling and spacing factor 1 (*RSF-1*) and β-catenin manifested restored sensitivity to paclitaxel in LC and LUAD, respectively (Chen et al., 2017; Duan et al., 2019). The latter also revealed the impact of β-catenin on the cascade regulation of miR-421 and KEAP1 in paclitaxel resistance. Several studies reported that CRISPR system-mediated modifications of multiple resistance-related genes resulted in the alleviated resistance to cisplatin, including excision repair cross-complementation group 1 (*ERCC1*), ribosomal protein S6 kinase 4 (*RSK4*), *NRF2*, and nicotinamide nucleotide transhydrogenase (*NNT*) in NSCLC (Heyza et al., 2019; Chrysostomou et al., 2021; Banas et al., 2022; Xu et al., 2023). Disrupting *NRF2* impaired A549 cell proliferation and increased chemosensitivity to cisplatin and carboplatin (Bialk et al., 2018). Besides, depletion of Pik3ca and Pik3cb subunits through CRISPR approaches reestablished the drug sensitivity to colchicine, paclitaxel and mitoxantrane in multidrug resistance cells of NSCLC (Zhang et al., 2020).

Fusion gene *AGK-BRAF* constructed with CRISPR approach promoted the phosphorylation of BRAF, MEK1/2, ERK1/2, and signal transducer and activator of transcription 3 (STAT3) and conferred resistance to the third-generation EGFR TKI, osimertinib (Vojnic et al., 2019). In addition to *AGK-BRAF*, fusion genes *CCDC6-RET*, *ESYT2-BRAF*, *FGFR3-TACC3*, and *EML4-ALK* have also been found to reduce sensitivity to osimertinib in *EGFR* del19-mutated LUAD cell line (Kobayashi et al., 2022), and fusion gene *EZR-ROS1* rendered resistance to ROS1 TKIs in HBECp53 cells (Sato et al., 2020). In BRAFV600E-driven lung tumors, knockout of RNA-binding motif single-stranded-interacting protein 3 (*RBMS3*) facilitated resistance to the treatment of BRAFV600E inhibitor dabrafenib plus MEK inhibitor trametinib (Vaishnavi et al., 2022). Resistance to PARP inhibitors was obtained by silencing schlafen 11 (*SLFN11*) using CRISPR/Cas9 technology in patient-derived xenograft model of small-cell lung cancer (SCLC) (Lok et al., 2017). The further knowledge of therapy resistance-associated genes through the use of CRISPR technology will contribute to precise treatment on patients with lung cancer and surmount resistance challenges.

### 3.4 CRISPR-barcoding

Genome instability and genetic heterogeneity render tumor evolution and intervention resistance (Guernet et al., 2016). DNA barcoding is a species identification system based on short DNA sequences (Savolainen et al., 2005). Combination of CRISPR/Cas9 technology, tumor barcodes, and high-throughput barcode sequencing (Tuba-seq) allows accurate quantification of the tumor size and potent study of multiple gene changes (Guernet et al., 2016; Rogers et al., 2017; Rogers et al., 2018). CRISPR-barcoding can be used to simulate various resistance mechanisms of lung cancer and assess the effectiveness of combination therapies. Furthermore, it allows the analysis of multiple genetic modifications, and it can also be used to trace and classify different subsets of cancer cells (Guernet et al., 2016). Additionally, the combination of CRISPR-barcoding with Tuba-seq allows the robust quantification of the effects of multiple tumor suppressor genes on tumor growth and their corresponding therapeutic responses in LUAD (Rogers et al., 2018; Cai et al., 2021; Foggetti et al., 2021). Recently, the integration of Tuba-seq into CRISPR-mediated multiplexed screens revealed that HRAS and NRAS presented a suppressive effect on *KRAS*-driven lung cancer growth *in vivo* (Tang et al., 2023).

As an innovative approach, CRISPR-barcoding/Tuba-seq exhibits a great potential for *in vivo* research on lung cancer characterized by multiple lesions, due to its advantages of precise quantification of tumor growth. It is expected to advance the investigation of intricate gene interaction networks within tumors (Rogers et al., 2017). Nevertheless, CRISPR-barcoding has its own limitations. Due to the inherent gene knockout nature of CRISPR/Cas9, CRISPR-barcoding exhibits certain limitations on the quantification of the oncogenic impact on tumor growth, but no limitations on the study of tumor suppressor genes. Besides, compared to conventional high-throughput CRISPR screening, currently, CRISPR-barcoding based screens only allows the study of a smaller number of genes in a single experiment. Despite these present limitations of CRISPR-barcoding, they are expected to be ultimately surmounted (Rogers et al., 2017), and it will facilitate the research on tumor development and genotype-specific therapeutic responses, eventually contributing to precise and personalized therapy on patients.

## 4 CRISPR-based mouse models of lung cancer

As a method of study, animal model is a bridge connecting preclinical basic research with clinical practices (Böck et al., 2014), which is the gateway of laboratory research to the clinical management. The earliest mouse model of cancer is subcutaneous or orthotopical transplantation of tumor cells derived from human or mice, enabling rapid and simple model establishment. However, cancer cell inoculation models do not represent normal morphology and heterogeneity of human disease (Kersten et al., 2017). Classic genetically engineered mouse models (GEMMs) can achieve *de novo* tumorigenesis and faithfully mimic human tumors, and thus it is extensively used in oncology research. However, high cost and time-consuming nature of GEMMs impede their applications and limit the scale of experimental studies (Kersten et al., 2017; Lundin et al., 2020). Conventional gene editing techniques including ZNFs and TALENs are faced with the difficulties in targeted modifications of multiple genes in animals due to the complexity of design and the different editing efficiency (Wang et al., 2013). In order to improve the effectiveness and representativeness of the complex animal models of cancer, novel approaches are needed.

CRISPR/Cas9-based genome editing completed the one-step generation of mice harboring multiple gene mutations by zygote injection (Wang et al., 2013). Over the past decade, the studies on CRISPR-based disease models have thrived, encompassing investigations into lung cancer model (Platt et al., 2014; Xue et al., 2014; Mou et al., 2015; Hartmann et al., 2021; Deng et al., 2022; Thege et al., 2022). Activation mutation of *KRAS* G12D and loss-of-function mutation of *p53* and *Lkb1* were realized in the Cre-dependent Cas9 mouse through CRISPR gene engineering, resulting in the development of invasive LUAD in less than 2 months (Platt et al., 2014). A recent comparative study has revealed that CRISPR-edited tumor model and classic GEMMs exhibit comparable tissue histopathological features and molecular expressional profiles including AP-1 transcription factor family-associated members, NOTCH1, NOTCH3, cMYC and squamous differentiation marker ΔNp63 (Hartmann et al., 2021). Moreover, this study utilized dual adeno-associated viral vectors to achieve inducible tumor formation at various stages in the context of constitutive Cas9-independent mice, thus avoiding the potential risks of off-target effects and inflammatory responses associated with Cas9 and Cre enzymes. Moreover, the emergence of ObLiGaRe doxycycline inducible SpCas9 (ODInCas9) mice enabled controllable repeated induction of Cas9 *in vivo* and modeling in less than 12 weeks (Lundin et al., 2020). NSCLC generated in these mice reflects the characteristics of human NSCLC, and exhibits an apparent response to chemotherapy and pathway inhibitors. The ODInCas9-based tumor models display the increased safety, cost, and efficiency of experiments, relative to Cas9-constitutively expressing models and conventional GEMMs. CRISPR lung cancer models have been widely utilized to validate cancer-associated genes, such as *Lkb1*, *SIK*, and *MYC* (Hollstein et al., 2019; Murray et al., 2019; Thege et al., 2022).

In addition, gene fusion caused by chromosomal rearrangements has been also elucidated in mouse models created by CRISPR technology. Two studies have reported the successful establishment of CRISPR-mediated *EML4-ALK* fusion gene-driven lung tumor models (Blasco et al., 2014; Maddalo et al., 2014), and an *RLF-MYCL*-induced metastatic SCLC mouse model (Ciampricotti et al., 2021). Considering the critical roles of these genetic alterations in promoting tumor initiation and progression and selecting targeted therapies, it is extremely urgent to develop effective animal models harboring these genetic changes for cancer research (Ferrara et al., 2020).

## 5 Delivery methods of CRISPR components

Physical methods including electroporation and microinjection are regularly used to delivery CRISPR components *in vivo* studies. Microinjection has the limitations of high costs and poor efficiency (Huang et al., 2022). However, an electroporation-based strategy transports Cas9/sgRNA ribonucleoproteins (RNPs) into mouse fertilized eggs with 100% efficiency, realizing extremely efficient gene editing *in vivo* (Chen et al., 2016). Notably, the world’s first human clinical trial based on CRISPR/Cas9 employed the technique of electroporation for the delivery of target plasmids, resulting in the generation of *PD-1* knockout T-cells (Lu et al., 2020). This trial suggests that it is safe and feasible for CRISPR-edited T-cells to treat advanced NSCLC patients. Another phase 1/2 trial in patients with metastatic NSCLC administering CRISPR/Cas9-edited T-cells is ongoing (Intima Bioscience, 2022).

Lentiviral (LV) vectors from a provirus of HIV have been widely used to deliver CRISPR/cas9 for target genes editing and library screening with a advantage of persistent gene transfer (Lino et al., 2018). Adenoviruses (ADVs) are characterized by large packaging capacity, high immunogenicity, limited cell types, and tissue specificity (Platt et al., 2014). Due to their efficient infection ability towards the lung epithelium in adult mice, ADVs are regarded as an ideal method for constructing the aforementioned lung cancer model harboring *EML4–ALK* inversion (Maddalo et al., 2014). Adeno-associated viruses (AAVs) are less immunogenic compared to other viral vectors, exhibiting clinical therapeutic promise (Huang et al., 2022). Transporting a single AAV vector with multiple elements including Cre to Cas9-constitutively expressing mice results in successful development of LUAD with *KRAS*, *p53*, and *LKB1* variants (Platt et al., 2014).

Considering that physical methods can only be applied *in vitro*, and that viral vectors have high immunogenicity and the risks of unexpected variations (Wang et al., 2022; Li et al., 2023), chemical approaches with high safety, convenience, and robust loading capacity need to be further developed. In recent years, numerous chemical strategies have been developed including liposome, nanoparticles, and polymeric carriers. A liposome-coated protamine sulfate-condensed non-viral vector delivered CRISPR plasmid into nucleus of tumor cells, subsequently causing the disruption of mutT homolog1 (*MTH1*) gene, eventually successfully mitigating NSCLC growth and metastasis and promoting tumor cell apoptosis (Wang et al., 2022). A method named selective organ targeting (SORT) using lipid nanoparticles was designed to specifically modify various tissues including lung (Cheng et al., 2020). This is the first rational design of nanoparticles targeting specific tissues. Besides, the direct delivery of multifunctional CRISPR/Cas9 RNPs by a carrier-free delivery system enables self-assembly of RNP complex, and this delivery method exhibits extensive potential in anti-KRAS therapy of lung cancer (Kim et al., 2018).

Currently, researchers have constructed nanoparticles with a library of biodegradable ionizable lipids for pulmonary mRNA delivery, which can achieve multiple intratracheal dosing (Li et al., 2023). Furthermore, using *Lactobacillus* rhamnosus GG (LGG) that can penetrate the tumor center, a novel self-driven delivery platform was established, and this platform exhibited a high efficiency in transporting CRISPR nanosystem (Yu et al., 2022). This strategy was used to knock down indoleamine 2,3-dioxygenase-1 for reversing tumor immunosuppression, and it proved to be a potent measure for gene therapy *in vivo*. The *survivin* gene expressed in most cancer is associated with inhibiting apoptosis and is a potential target to gene therapy. In a recent study of delivery using polymer carriers in lung cancer, a technology combining mannose functionalized four-arm polyglycidyl methacrylate cationic polymers and pCas9-survivin was employed for plasmid DNA delivery (Wang et al., 2023). The GM/pCas9-survivin can be recognized by mannose receptors with higher expression in tumor cells than in healthy cells. Binding of GM/pCas9-survivin to the mannose receptor allows for selective entry of DNA into lung cancer cells and knocking out survivin gene efficiently (Wang et al., 2023).

## 6 Discussion

Over the past decade, CRISPR-based genome editing technology has developed rapidly. CRISPR technology plays an increasing important role in advancing our understanding of lung cancer biology and in diagnosis and therapy of lung cancer. The precision and multi-functionality of CRISPR/Cas systems allow targeted manipulation of oncogenes, tumor suppressor genes, and genes associated with therapy resistance. This has significantly expanded our ability to decipher the genetic and molecular mechanisms of lung cancer, paving the way for the identification of novel therapeutic targets and predictive biomarkers.

CRISPR-based methods exhibit great potentials in early lung cancer screening and prognostic assessment, particularly in detecting disease-specific biomarkers such as cfDNA, miRNAs, and plasma proteins. These methods are expected to improve prognosis by implementing timely interventions and personalized treatment regimens. Moreover, CRISPR technology has also significantly advanced the development of animal models for lung cancer. Although there are significant differences between the immune system, metabolic function, and stress response of mice and humans, the contribution of experimental mice to medical research is enormous. Further research is necessary to fully understand the distinctions between the two species and how human diseases can be accurately modeled under the most suitable and effective conditions (Rydell-Törmänen and Johnson, 2019). The establishment of CRISPR-edited mouse models including advanced inducible models provides more accurate, rapid, and secure representations of human lung cancer, facilitating the preclinical animal evaluation of therapeutic interventions.

However, CRISPR does present some disadvantages. One of the primary shortcomings is the off-target effects, which could cause unpredictable outcomes. This is also a considerable issue facing gene editing to treat disease. Continued animal research and technological development will minimize the risks of CRISPR gene editing technology and promote its clinical translation. Strategies to mitigate the off-target effects are being studied including Cas9 protein and sgRNA improvement (Manghwar et al., 2020; Naeem et al., 2020; Guo et al., 2023). In addition, the limitations of delivery methods are crucial factors affecting its safety and effectiveness.

As we look to the future, further refinement of CRISPR technology and the development of innovative delivery methods, such as non-viral chemical carriers, will be essential for its applications. Addressing the challenges related to off-target effects, immunogenicity, and safety concerns will contribute to ensuring the clinical feasibility and broad applicability of CRISPR-based therapies in lung cancer and beyond. Additionally, collaboration between researchers, clinicians, and biotechnologists is important for fully exerting the potential of CRISPR technology and promoting clinical management of patients.
